# The Efficacy of Paraprobiotic Lozenges (*Lactobacillus helveticus* MIMLh5) for the Prevention of Acute and Chronic Nose and Throat Infections in Children

**DOI:** 10.3390/medicina60081235

**Published:** 2024-07-30

**Authors:** Ivan Baljošević, Vladan Šubarević, Katarina Stanković, Aleksandra Bajec Opančina, Mladen Novković, Masa Petrovic

**Affiliations:** 1 Department of Pediatric Otorhinolaryngology, Institute for Mother and Child Health Care of Serbia Dr. Vukan Čupić, 11070 Belgrade, Serbia; 2 Faculty of Medicine, University of Belgrade, 11000 Belgrade, Serbia; 3 Institute for Cardiovascular Diseases “Dedinje”, 11000 Belgrade, Serbia

**Keywords:** paraprobiotics, prevention, chronic infections

## Abstract

*Background and Objectives:* Tonsillitis is common in children and is predominantly caused by viruses and, less frequently, by bacteria such as group A beta-hemolytic streptococcus. The treatment primarily involves supportive care; however, the overuse of antibiotics remains a concern due to rising antibiotic resistance. Probiotics, particularly Lactobacillus strains, have been shown to modulate immune responses, offering a potential alternative treatment. *Materials and Methods:* Our prospective single-arm, open-label study included 98 pediatric patients aged 5–15 years with recurrent throat and tonsil infections, from October 2022 to January 2023. Patients received lozenges containing heat-killed *Lactobacillus helveticus* MIMLh5. Monthly follow-ups involved a comprehensive ear, nose, and throat examination, throat cultures, and recording the frequency of infections and antibiotic use. Data were analyzed using SPSS 29.0, with statistical significance set at *p* < 0.05. *Results:* After three months, significant reductions were observed in the occurrences of nose and throat infections (*p* < 0.001), enlarged submandibular glands (*p* < 0.001), and positive throat cultures (*p* < 0.001). Antibiotic and corticosteroid prescriptions also significantly decreased (*p* < 0.001). Among children aged 5–10 years, significant improvements were noted in throat and tonsil infections (*p* < 0.001) and positive throat cultures (*p* = 0.012). Overall, there was a substantial reduction in school days missed (*p* < 0.001). *Conclusions:* The use of paraprobiotic *Lactobacillus helveticus* MIMLh5 lozenges significantly reduced the incidence of recurrent throat and tonsil infections in children, decreased the need for antibiotics and corticosteroids, and improved overall clinical outcomes without adverse effects. These findings support the use of paraprobiotic supplements as a safe and effective preventive measure for pediatric throat and tonsil infections.

## 1. Introduction

Palatine or faucial tonsils and adenoid vegetations (nasopharyngeal tonsil) represent lymphatic tissue located in the lateral oropharynx (tonsillar fossa) and posterior part of the nasopharynx, respectively [1]. This lymph tissue is very similar to the tissue found in the lymphatic glands or nodes, which are localized in the neck or other parts of the body. The tonsils and adenoids comprise part of Waldeyer’s ring, a lymphatic structure that plays a crucial role in the immune system, which also includes the tubal and lingual tonsils. Waldeyer’s ring acts as the first line of defense against infections and carries out the antigenic stimulation of environmental factors [2].

Tonsillitis is the inflammation of the tonsils and is a very common infection among children but extremely rare in the pediatric population younger than two years of age. Tonsillitis can be attributed to a variety of microorganisms including viruses and bacteria; however, viruses are more common in toddlers, while in the remainder of the pediatric population, streptococci are cited as the most common causal agent. Nearly 80% of viral tonsillitis cases are caused by the adenovirus, rhinovirus, influenza, coronavirus, and respiratory syncytial virus. Less frequently, Epstein–Barr virus, Herpes simplex virus, cytomegalovirus, or HIV could also be a culprit. With regard to bacterial tonsillitis, the most common strain is beta-hemolytic streptococcus group A (GAHBS), followed by Staphylococcus aureus (including methicillin-resistant Staphylococcus aureus or MRSA), Streptococcus pneumoniae, Mycoplasma pneumoniae, and Chlamydia pneumoniae [3]. It is important to note that in approximately a third of cases, GAHBS and influenza virus may be isolated together in pharyngotonsillitis as a co-infection.

In the majority of cases, tonsillitis is a self-limiting disease. As the most frequent pathogens are viruses, the main treatment of acute tonsillitis is based on supportive care, including analgesia with NSAIDs and hydration [4,5]. Consequently, tonsillitis has a severe economic burden, with an annual 35,000,000 days lost from work or school attributed to tonsillitis [6].

It is important to emphasize that most pathogens responsible for tonsillitis belong to healthy oral microbiota and thus do not require full eradication [7]. Therefore, it is important to consider the risks and benefits when considering the inclusion of antibiotics in the treatment of tonsillitis. The risks of antibiotic use include increased antibacterial resistance, GI upset, diarrhea, Clostridioides difficile infection, and cost [8]. While antibiotics may reduce complications and symptom duration, the effect is small. Additionally, antibiotics can be useful in high-risk populations of rheumatic heart disease and rheumatic fever [9]. A recent study has observed that general practitioners often resort to prescribing broad-spectrum antibiotics for patients diagnosed with tonsillitis or pharyngitis, irrespective of the disease’s viral etiology. This practice has resulted in tonsillitis being identified as having the highest rate of potentially inappropriate treatments (46%) within primary healthcare settings [10].

Probiotic bacterial cells (either viable or inactivated), on the other hand, may have a huge impact on cells involved human immunity, for example, dendritic cells, monocytes, natural killer cells, macrophages, lymphocytes, and epithelial cells. Moreover, immune regulation is one of the main benefits of probiotic bacteria mainly due to the release of the anti-inflammatory cytokine. Widely adopted probiotics in commercial products are lactobacilli and bifidobacteria, but the yeast Saccharomyces cerevisiae is also commonly employed. Specific strains of Lactobacillus may modulate cytokine production by immune cells, and Bifidobacterium induces tolerance acquisition [11].

Lactobacilli, microorganisms that have long been integrated into the human body, are often considered “old friends” by their hosts. These bacteria are not only benign but are also essential modulators of the human immune system. However, recent decades have witnessed a decline in these “old friends” due to significant environmental shifts, akin to those we are experiencing currently [12]. This change threatens to disrupt the millennia-old symbiotic balance between certain bacterial species and the human ecosystem. Increasing evidence has suggested that strain *Lactobacillus helveticus* MIMLh5 exhibits probiotic properties with health-promoting attributes [13,14,15].

The aim of our study was to evaluate the efficacy of lozenges containing inactivated (heat-killed) bacteria on the probiotic strain *Lactobacillus helveticus* MIMLh5 in preventing acute and chronic nose and throat infections in children with a history of recurrent infections.

## 2. Materials and Methods

This prospective, single-arm, open-label study enrolled 100 consecutive patients, aged between 5 and 15 years, presenting electively due to chronic, recurrent infections (more than 3 infections annually with symptoms lasting for longer than 2 weeks) of the throat and tonsils who sought care at the Ear, Nose, and Throat department of Institute for Mother and Child Health Care of Serbia Dr. Vukan Čupić, a tertiary pediatric institution located in Belgrade, Serbia. The inclusion period spanned from 1 October 2022 to 31 January 2023. Exclusion criteria encompassed patients with syndromes, immunodeficiency, or any other associated diseases. For the duration of the study, two patients were lost to follow-up. Ethical approval for this study was granted by the Institutional Review Board (No. 8/169).

At the first visit, a comprehensive ear, nose, and throat examination was performed by the physician who also concurrently completed a study questionnaire noting the patient’s age, gender, number of infections of the nose and pharynx and throat and tonsils in the past year, enlargement of the submandibular glands, and previous use of antibiotics and corticosteroids for the treatment of a throat and tonsil infection. Additionally, the physician also took a throat culture during the examination and later noted the results of the culture at the examination as well as the causative agent for positive cultures. Patients who fit the inclusion criteria, along with their guardians, received verbal information on the administration of the oral paraprobiotic lozenge and subsequently gave verbal and written informed consent to participate in the study. Both patients and their guardians were instructed that the lozenge, formulated as a slow-dissolving orosoluble tablet, should be taken immediately before bedtime. Furthermore, patients were advised to refrain from consuming any liquids for at least two hours subsequent to the tablet’s ingestion. Throughout the duration of the use of the paraprobiotic lozenges, no adverse effects were reported by the patients.

After the initial visit, the children were monitored every month. During the follow-up visits, a comprehensive ear, nose, and throat examination was performed, including a throat culture, and the physician concurrently completed a follow-up examination study questionnaire noting if the patient had infections of the nose and pharynx and throat and tonsils in the past month, whether antibiotics were administered in the past month, and the results of the throat culture as well as the causative agent for positive cultures from the follow-up examination. The same data were recorded during each follow-up appointment.

The results are presented as count (%), means ± standard deviation, or median (25th–75th percentile) depending on data type and distribution. Analysis to determine the efficacy of treatment included parametric (*t*-test, one-way ANOVA) and non-parametric tests (Cochran’s Q test, McNemar’s test). All *p*-values less than 0.05 were considered significant. All data were analyzed using SPSS 29.0 (IBM Corp. Released 2022. IBM SPSS Statistics for Windows, Version 29.0. Armonk, NY, USA: IBM Corp.).

## 3. Results

This study involved 98 pediatric patients with chronic, recurring infections in the nose, pharynx, throat, and tonsils, with 48 (49.0%) being male. Among them, 69 (70.4%) were aged 5–10 years, and 29 (29.6%) were aged 10–15 years.

After three months of treatment with the paraprobiotic *L. helveticus* lozenges, there was a statistically significant reduction in all noted positive clinical findings (Table 1). The overall occurrences of infections of the nose and pharynx and throat and tonsils were significantly reduced (*p* < 0.001), together with a notable decrease in enlarged submandibular glands (*p* < 0.001), subsequently leading to a considerable drop in positive throat cultures (*p* < 0.001). Both antibiotics and corticosteroids were significantly less frequently prescribed (*p* < 0.001).

In children aged 5–10 years, a statistically significant improvement in the rate of positive clinical findings was observed. The incidence of throat and tonsil infections was significantly reduced (*p* < 0.001), with a noticeable decrease in the size of swollen submandibular glands (*p* = 0.012), as well as positive throat cultures (*p* = 0.012). The occurrences of infections in the nose and pharynx did not exhibit a statistically significant decrease (*p* = 0.070). Neither antibiotics nor corticosteroids were prescribed less frequently.

Furthermore, throat cultures yielding positive results were substantially less prevalent throughout the therapy with paraprobiotic lozenges compared to the previous year before the lozenges were implemented. This effect was noted in all throat cultures, with no obvious variation in the occurrences among particular bacteria. (Table 2). Additionally, in subjects aged 5–10, similar findings were noted regarding positive throat cultures (Table 2). Overall, the frequency of positive results from throat cultures was considerably lower during the treatment with paraprobiotic lozenges compared to the year prior to the introduction of the medicine.

When comparing all subjects, as well as the study population split into two groups, ages 5 to 10 and ages 11 to 15 (Table 3), there were no statistically significant disparities in the gender distribution, duration of fever, or presence of positive throat cultures either prior to or following treatment. Except for the second month of observation, there were no noticeable differences in terms of school absences or days with fever. Notably, participants had a significant reduction in the number of school days missed when using the lozenges during the three-month follow-up period (*p* < 0.001).

## 4. Discussion

The results of this study demonstrated how the treatment with the paraprobiotic agent, *Lactobacillus helveticus* MIMLh5, significantly lowered the number of nasal and throat infections, as well as infections of the throat and tonsils, when administrated to children presenting recurrent infections of the throat and tonsils.

Probiotics are living microorganisms that could help maintain the normal microbiota of the body, including the gastrointestinal tract, thus contributing to overall health. Research findings from the literature emphasize the importance of probiotics in modulating immune responses, providing protection against arthritis and intestinal inflammation, and enhancing resistance to diseases such as diabetes [6]. Although extremely interesting, the use of probiotics is demonstrating some drawbacks. For instance, probiotics should be formulated in such a way that they can reach the target site in the host after surviving throughout processing, storage, and gastrointestinal transit while remaining highly viable and in sufficient numbers [16]. Moreover, the adequate intake amount of live microorganisms is still a matter of discussion [17,18]. All these drawbacks related to the administration of viable microorganisms led to the interest in non-viable probiotic preparations, termed paraprobiotics or postbiotics [19]. Paraprobiotics have been proven to modulate anti-inflammatory and positive immune responses. Bacteria-killed cells demonstrated significant safety if compared to probiotics, i.e., a reduced risk of sepsis and antibiotic resistance, as well as technological and practical benefits, i.e., longer shelf life, since the cold chain is not required for microorganism viability and stability [20,21]. Additionally, another great advantage is no loss of bioactivity when administered in combination with antibiotics or antifungal agents [22].

Lactobacilli are Gram-positive bacteria composed of dozens of species that inhabit various environments with high levels of soluble carbohydrates, protein breakdown products, vitamins, and low concentrations of oxygen. Some lactobacilli are native residents of the human gastrointestinal tract, where they are considered beneficial components of the microbiota.

*Lactobacillus helveticus* can effectively antagonize group A streptococci and modulate the immune response in epithelial cells, dendritic cells, and macrophages. Thanks to its surface protein SlpA, it exhibits anti-inflammatory effects by reducing NF-kB activation and stimulating innate immune system effects through the expression of pro-inflammatory factors such as tumor necrosis factor-alpha and COX-2 in human macrophage cell lines [7,8]. *Lactobacillus helveticus* is a potential pharyngeal probiotic due to its ability to adhere to human epithelial cell lines and effectively antagonize group A streptococci on these cells. The immunostimulatory properties of *L. helveticus* are primarily mediated by its S-layer protein [5,9]. Studies on *Lactobacillus helveticus*, Lacticaseibacillus casei, and Lacticaseibacillus rhamnosus have induced early pro-inflammatory cytokines such as IL-8, TNF-alpha, IL-12, and IL-6. NF-kB- and TLR2-dependent signaling are in turn increased with treatment with these probiotics, further demonstrating their immunostimulatory effect [10]. In our study, throat swabs were analyzed before and after three months of taking slow-dissolving orosoluble tablets with tyndallized *Lactobacillus helveticus* MIMLh5 cells. We found a statistically significant reduction in the number of positive throat swabs in children who took the medication for three months.

In a multicenter, double-blind, placebo-controlled trial conducted in France from December 2006 to March 2007, children aged from 3 to 7 years old were randomized to either a daily synbiotic (*Lactobacillus helveticus* R0052, *Bifidobacterium longum* supsp. *infantis* R0033, *Bifidobacterium bifidum* R0071, and *fructooligosaccharides*) or placebo supplementation for 3 months. The study included only the winter period. This study indicated a clinically relevant beneficial effect of the three-month use of synbiotic with *Lactobacillus helveticus* in preventing common acute infectious diseases of the ear, throat, and nose in school-age children from 3 to 7 years old and limiting the risk of school day loss [23]. Our study showed a significant reduction in the number of school days missed when using the lozenges during the three-month follow-up period which can be also an indicator of the severity of the clinical presentation of infection.

In the prospective study conducted by Cavaliere et al., involving 22 adults and 16 children with a history of recurrent rhinopharyngitis (at least three episodes in the year preceding the study), participants were treated with one buccal tablet (*L. helveticus*) daily for 90 days [24]. Clinical symptoms were assessed before and after treatment using a 20-item sinonasal outcome test (SNOT-20) and Visual analog scales (VASs) for nasal obstruction, headache, anosmia, nasopharyngeal drainage, sneezing, and cough. Both adult and pediatric patients showed a significant reduction and improvement in VAS scores for all assessed symptoms except for nasopharyngeal drainage in adults and headache in children. The number of muciparous cells and neutrophils in nasal swabs, as well as inflammatory indices in the blood, were reduced. In children, the incidence of positive throat swabs was lower after treatment [24].

The recommendation for corticosteroid use in the pediatric population for upper respiratory tract infections is weak; however, sore throat is a common condition associated with a high rate of antibiotic and corticosteroid prescriptions, despite limited evidence for the effectiveness of antibiotics or corticosteroids [25]. Corticosteroids are unlikely to reduce the recurrence or relapse of symptoms or days missed from school or work. A systematic review of using probiotics for the prevention of pediatric upper respiratory tract infections showed a good safety profile in childhood, and none of the studies reported any serious adverse events related to probiotics [26]. Both antibiotics and corticosteroids were significantly less frequently prescribed to our participants, which supported the statistically significant reduction in all noted positive clinical findings. Probiotics can play a significant role in immune stimulation and positively impact the reduction in various infections. The results of our study suggest that a paraprobiotic supplement containing *Lactobacillus helveticus* can be beneficially applied in the prevention of throat and tonsil infections. Continuous application for at least three months is recommended and results in significant improvements in reducing the incidence of throat and tonsil infections, as well as the use of antibiotics and corticosteroids in treatment. No adverse effects were registered, making the supplement completely safe for use in children.

This study has several limitations that should be noted. The prospective single-arm design restricts the ability to establish causality between the use of paraprobiotic lozenges and the observed outcomes, highlighting the need for randomized controlled trials with placebo control groups. The relatively small sample size from a single tertiary pediatric institution in Serbia limits the generalizability of our findings to broader populations. Additionally, this study’s follow-up period was limited, precluding the assessment of long-term effects. Future studies with larger, more diverse cohorts and extended follow-up periods are necessary to validate and extend our findings.

Moreover, the reliance on self-reported adherence and the open-label design introduce potential biases that could affect the results. Recall bias may have also influenced the number of infections reported by parents. Furthermore, we had no control over whether patients were taking any other supplements or medications that parents did not report during the study period, which could confound the results.

## 5. Conclusions

Our study demonstrated a significantly lower number of nasal and throat infections, as well as infections of the throat and tonsils, in children who used lozenges with heat-inactivated *Lactobacillus helveticus* MIMLh5. The enlargement of submandibular glands was found in a significantly smaller number of children compared to the period before the administration of medication in the past year.

## Figures and Tables

**Table 1:** The number of participants having positive clinical findings at baseline (during the past year) and at the three-month follow-up during the paraprobiotic lozenge treatment.

All Subjects						
Positive Clinical Findings	Baseline (Past Year)	During the Study	*p*-Value *	Follow-Ups during the Treatment		
				1st Month	2nd Month	3rd Month
Nose and Pharynx	94 (95.9%)	79 (83.2%)	<0.001	58 (59.2%)	52 (53.1%)	41 (41.8%)
Throat and Tonsils	94 (95.9%)	54 (54.5%)	<0.001	38 (38.8%)	22 (22.4%)	25 (25.5%)
Submandibular Glands	63 (64.3%)	47 (47.5%)	0.005	31 (31.6%)	19 (19.4%)	21 (21.4%)
Throat Culture	55 (56.1%)	23 (23.2%)	0.003	13 (13.3%)	6 (6.1%)	8 (8.2%)
Antibiotics Prescribed	98 (100.0%)	55 (55.6%)	<0.001	42 (42.9%)	26 (26.5%)	28 (28.6%)
Corticosteroids Prescribed	23 (23.5%)	14 (14.1%)	0.122	11 (11.2%)	9 (9.2%)	3 (3.1%)
**Ages 5–10 only**						
Nose and Pharynx	67 (97.1%)	61 (88.4%)	0.07	48 (69.6%)	40 (58.0%)	35 (50.7%)
Throat and Tonsils	65 (94.2%)	38 (55.1%)	<0.001	26 (37.7%)	19 (27.5%)	17 (24.6%)
Submandibular Glands	49 (71.0%)	37 (53.6%)	0.012	24 (34.8%)	17 (24.6%)	18 (26.1%)
Throat Culture	33 (47.8%)	16 (23.2%)	0.002	10 (14.5%)	4 (5.8%)	5 (7.2%)
Antibiotics Prescribed	69 (100.0%)	40 (58.0%)	N/A	31 (44.9%)	22 (31.9%)	18 (26.1%)
Corticosteroids Prescribed	19 (27.5%)	12 (17.4%)	0.189	10 (14.5%)	7 (10.1%)	2 (2.9%)

Data are presented as *n* (%); for Cochran’s Q test, all values were *p* < 0.001, * McNemar’s test presented in table. All values *p* < 0.05 were considered statistically significant.

**Table 2:** The number of participants with positive throat cultures at baseline (during the past year) and at the three-month follow-up during the *L. helveticus* paraprobiotic lozenge treatment.

All Subjects					
Positive Throat Cultures	Past Year (Before Treatment)	Follow-Ups While on Treatment			*p*-Value *
		1st Month	2nd Month	3rd Month	
Beta-hemolytic streptococci	32 (74.4%)	8 (61.5%)	3 (50.0%)	3 (37.5%)	
*Staphylococcus aureus*	7 (16.3%)	4 (30.8%)	3 (50.0%)	5 (62.5%)	
*Haemophilus influenzae*	4 (9.3%)	1 (7.7%)	0 (0.0%)	0 (0.0%)	
**Total**	43 (100%)	13 (30.2%)	6 (14.0%)	8 (18.6%)	<0.001
**Ages 5–10**					
Beta-hemolytic streptococci	25 (75.8%)	7 (70.0%)	3 (75.0%)	2 (40.0%)	
*Staphylococcus aureus*	5 (15.2%)	2 (20.0%)	1 (25.0%)	3 (60.0%)	
*Haemophilus influenzae*	3 (9.1%)	1 (10.0%)	0 (0.0%)	0 (0.0%)	
**Total**	33 (47.8%)	10 (30.3%)	4 (12.1%)	5 (15.2%)	<0.001
**Ages 11–15**					
Beta-hemolytic streptococci	7 (77.8%)	1 (33.3%)	0 (0.0%)	1 (33.3%)	
*Staphylococcus aureus*	1 (11.1%)	2 (66.7%)	2 (100.0%)	2 (66.7%)	
*Haemophilus influenzae*	1 (11.1%)	0 (0.0%)	0 (0.0%)	0 (0.0%)	
**Total**	9 (47.8%)	3 (14.5%)	2 (5.8%)	3 (7.2%)	<0.001

Data are presented as *n* (%). * Cochran’s Q test. All values *p* < 0.05 were considered statistically significant.

**Table 3:** Study population demographics, days missed of school, fever, positive throat cultures before and after study.

	Ages 5–10 (*n* = 69)	Ages 11–15 (*n* = 29)	All (*n* = 98)	*p*-Value
**Age**				
**Gender (male)**	34 (49.3%)	14 (48.3%)	48 (49.0%)	0.928 ^b^
**Days missed of school**				
M1	46 (66.7%)	16 (55.2%)	62 (62.6%)	0.281^b^
M2	35 (50.7%)	8 (27.6%)	43 (43.4%)	**0.035 ^b^**
M3	29 (42.0%)	11 (37.9%)	40 (40.4%)	0.706 ^b^
**Fever (yes)**				
M1	45 (65.2%)	15 (51.7%)	60 (60.6%)	0.211 ^b^
M2	34 (49.3%)	7 (24.1%)	41 (41.4%)	**0.021 ^b^**
M3	30 (43.5%)	11 (37.9)	41 (41.4%)	0.611 ^b^
**Duration of Fever (days)**				
M1	3.4 ± 1.5	4.4 ± 2.5	3.67 ± 1.81	0.070 ^a^
M2	3.1 ± 1.2	2.4 ± 1.2	2.95 ± 1.18	0.203 ^a^
M3	3.0 ± 1.6	3.2 ± 1.0	3.05 ± 1.48	0.733 ^a^
**Positive Throat Culture**				
In past year	33 (47.8%)	9 (31.0%)	43 (43.4%)	0.125 ^b^
During the study	16 (23.2%)	7 (24.1%)	12 (27.9%)	0.919 ^b^

All values presented as *n* (%) or mean ± SD. *p*-value < 0.05 was considered statistically significant. Tests: ^a^ *t*-test, ^b^ Chi-squared.

## Data Availability

The data presented in this study are available on request from the corresponding author due to privacy reasons.
